# Supplementary material for: ‘Druggability’ of the Per–Arnt–Sim (PAS) domains of human PAS domain kinase, a therapeutic target for metabolic and liver disorders
Source: QRB Discov. 2024 Feb 2;6:e18. doi: 10.1017/qrd.2024.1 (PMC12231308; doi:10.1017/qrd.2024.1)
Supplement: Xu et al. supplementary material [file S2633289224000012sup001.docx]

**Supplementary Information; Xu *et al.***


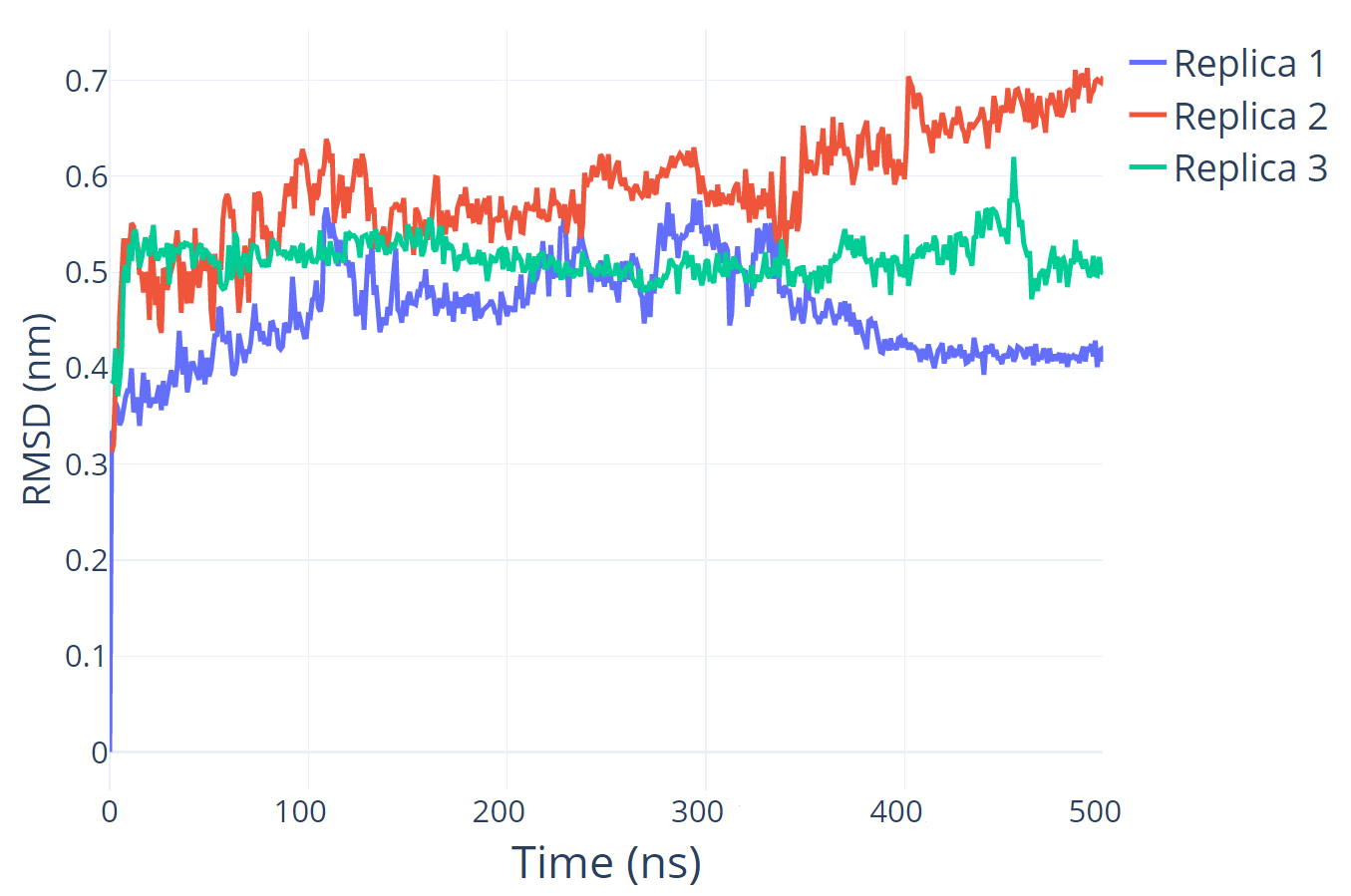


**Figure S1** Root mean square deviation (RMSD) of the PAS1 domain model, showing calculations for each replica. The RMSD fit was done over the backbone atoms and calculated for the whole protein (heavy atoms within the backbone and sidechains.


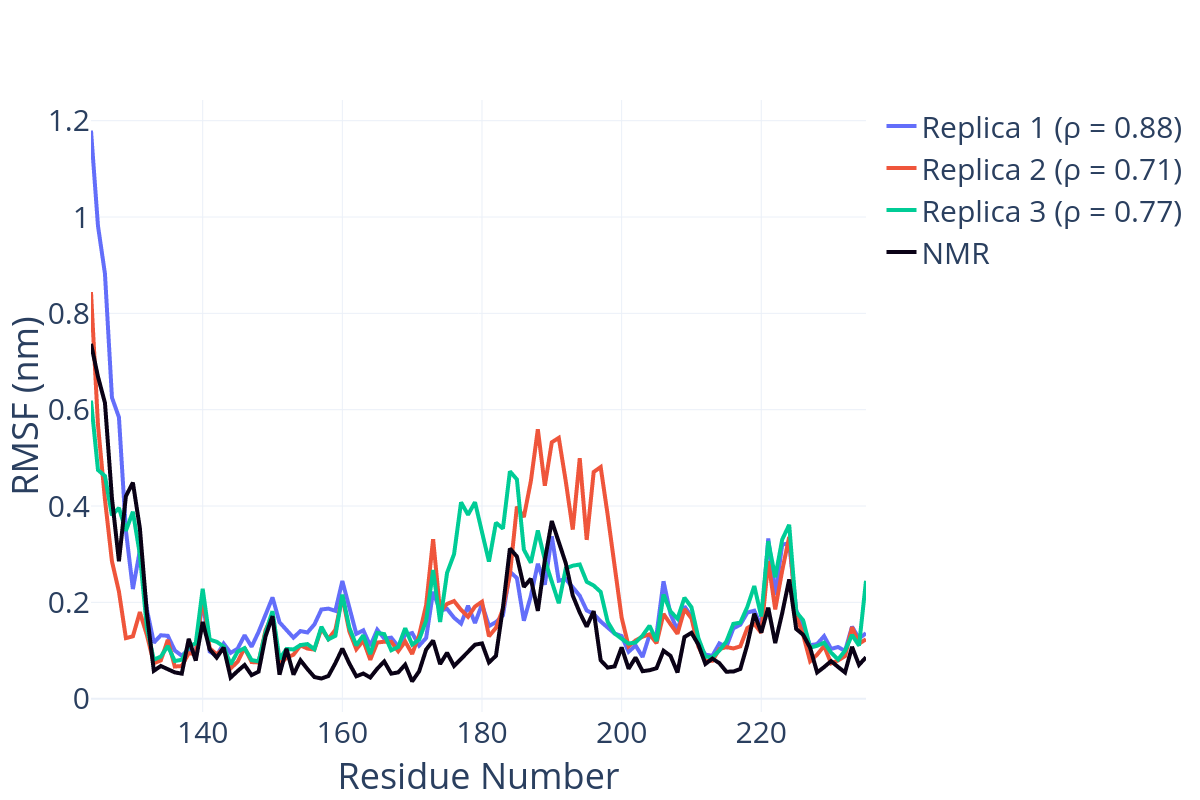


**Figure S2** Per-residue root mean square fluctuations (RMSF) calculated from 500 ns MD simulation for every replica of the PAS1 domain model, compared to the per-residue RMSF values obtained for the solution NMR ensemble (Amezcua et al., 2002; PDB code: 1LL8).


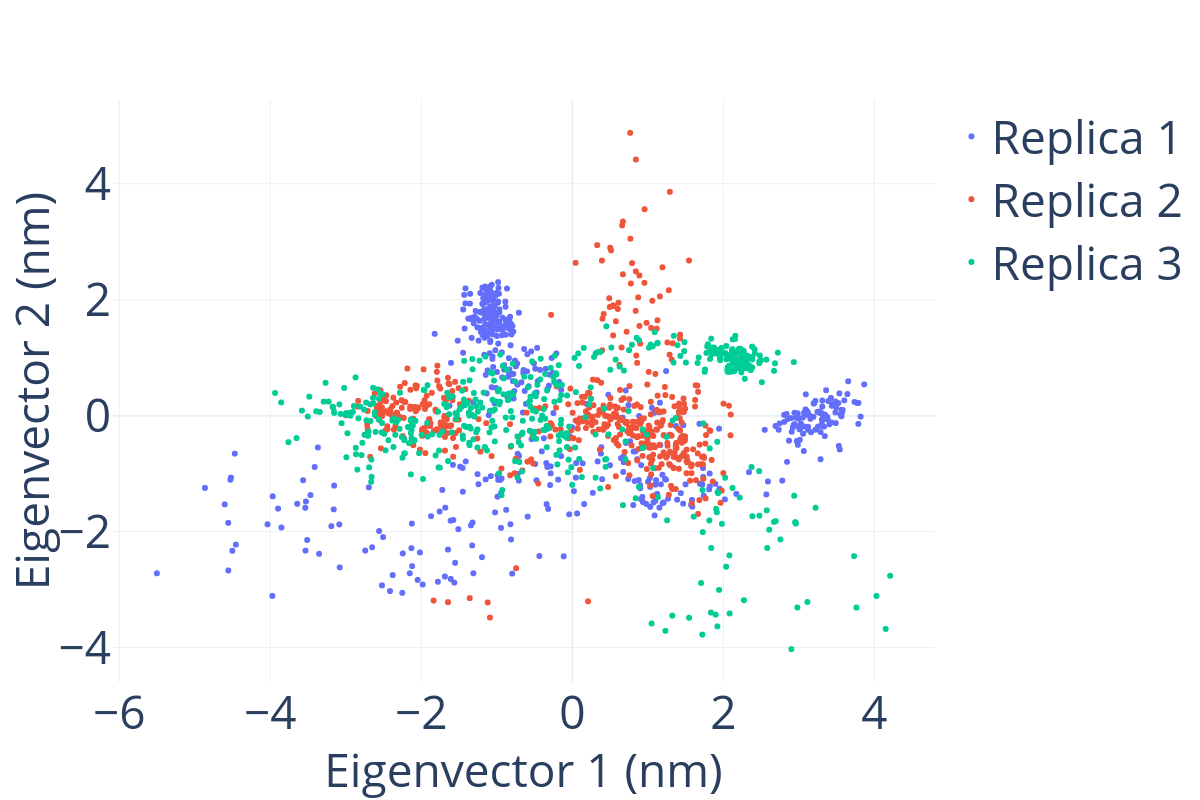


**Figure S3** Principal component analysis (PCA) of 500 ns MD simulation of the PAS1 domain model.


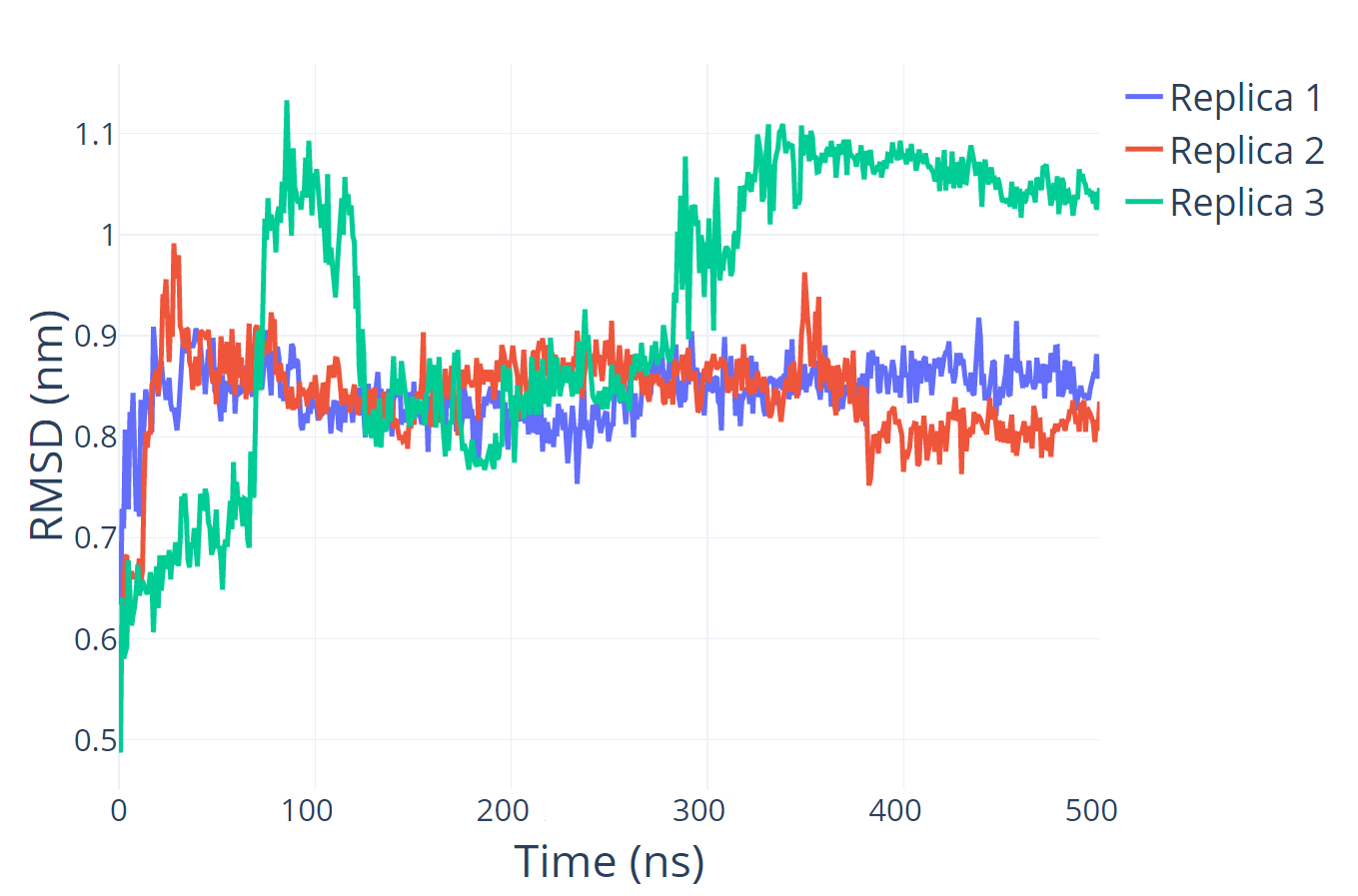


**Figure S4** Root mean square deviation (RMSD) of the PAS3 domain homology model, showing calculations for each replica. The RMSD fit was done over the backbone atoms and calculated for the whole protein (heavy atoms within the backbone and sidechains.


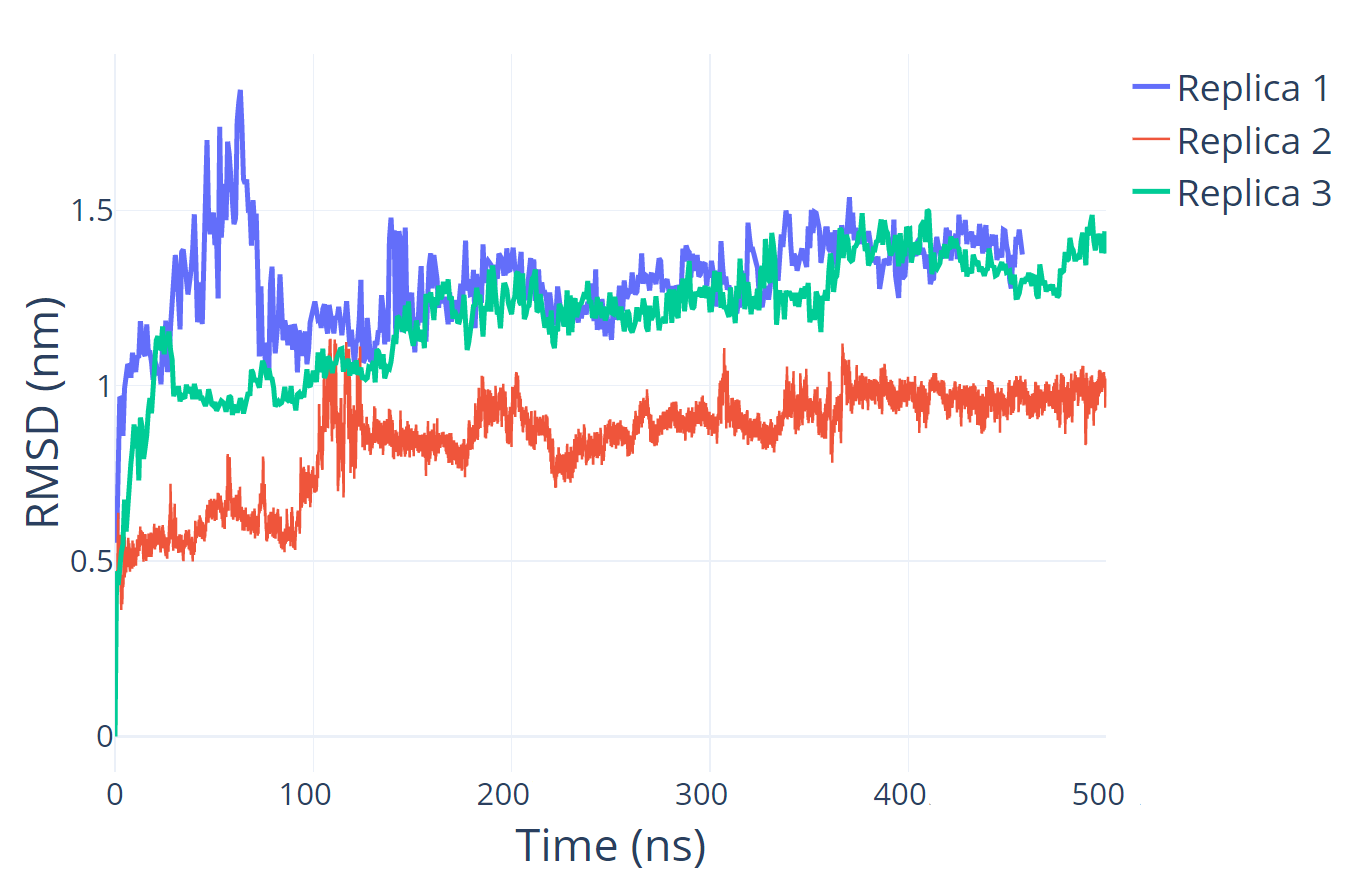


**Figure S5** Root mean square deviation (RMSD) of the PD4 domain homology model, showing calculations for each replica. The RMSD fit was done over the backbone atoms and calculated for the whole protein (heavy atoms within the backbone and sidechains. The domain unfolded in 2 out of 3 simulations (replica 1 and replica 3).


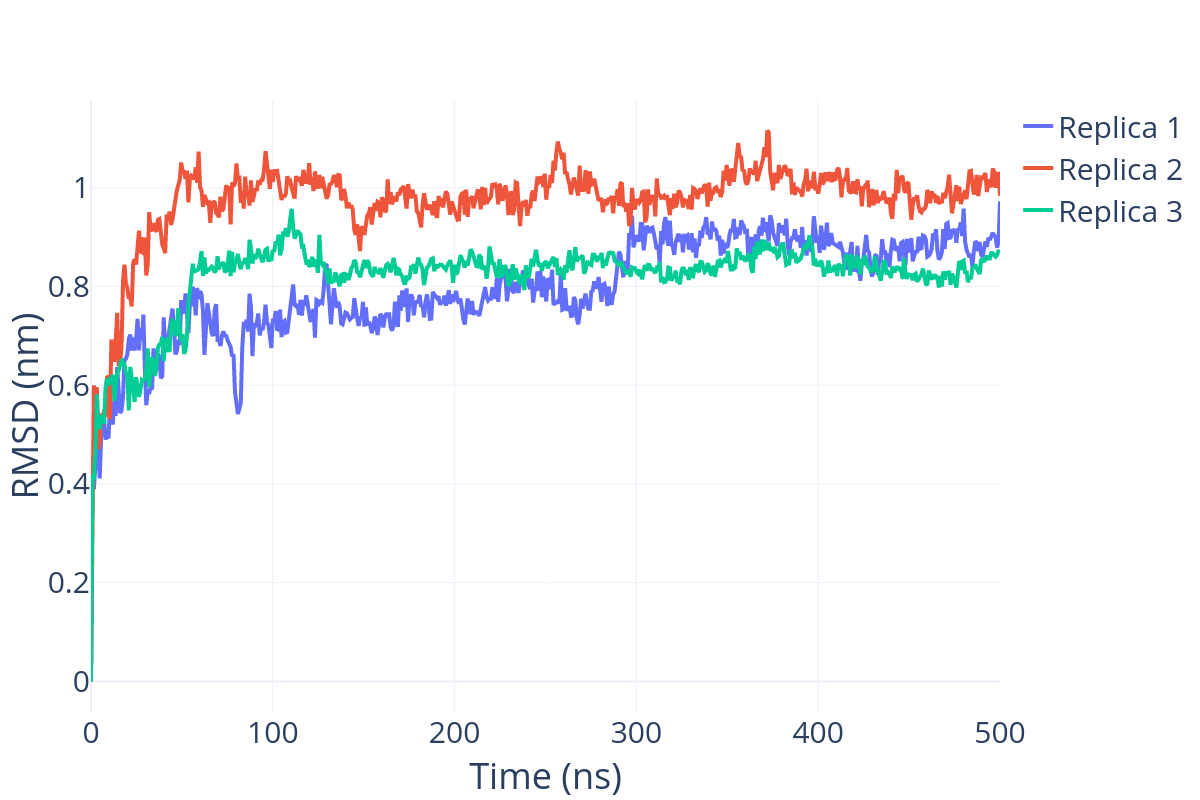


**Figure S6** Root mean square deviation (RMSD) of the PAS1-PAS2 segment of human PASK, showing calculations for each replica. The RMSD fit was done over the backbone atoms of the whole segment and calculated for the whole protein (heavy atoms within the backbone and sidechains.


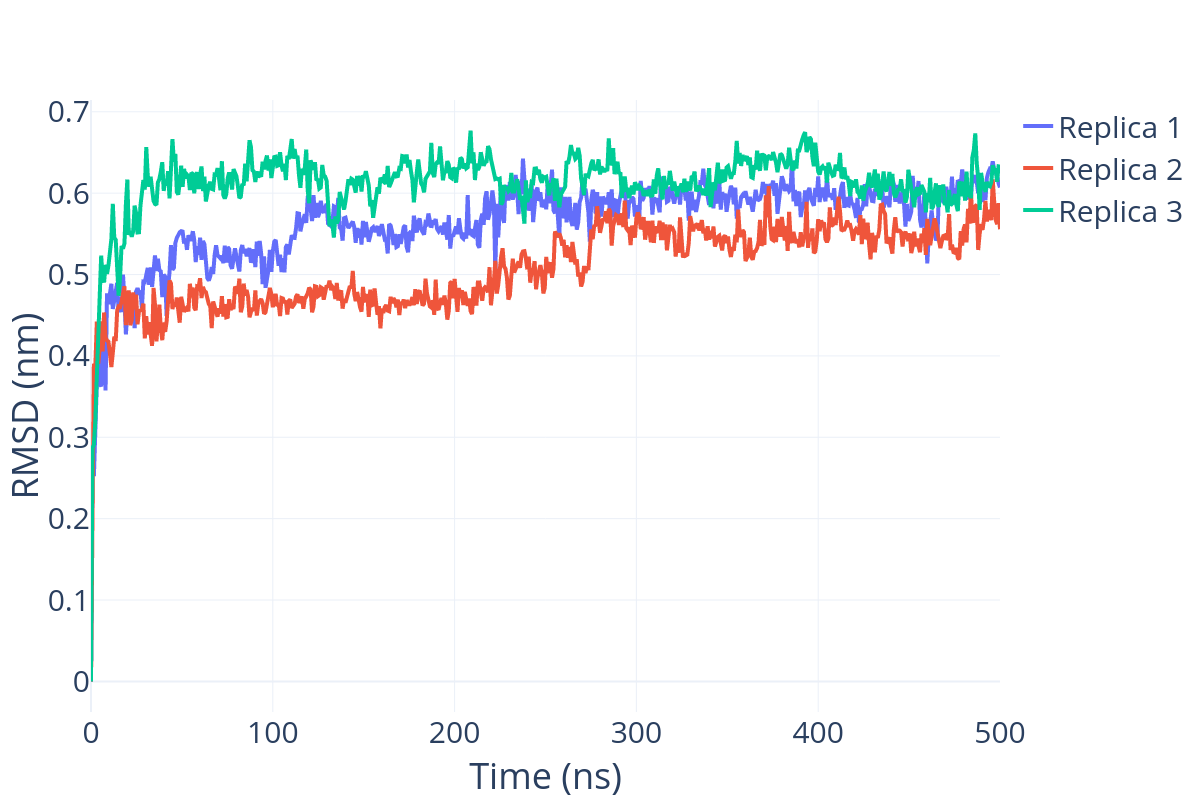


**Figure S7** Root mean square deviation (RMSD) of the PAS1 domain within the PAS1-PAS2 segment of human PASK, showing calculations for each replica. The RMSD fit was done over the backbone atoms of the whole segment and calculated for the whole protein (heavy atoms within the backbone and sidechains.


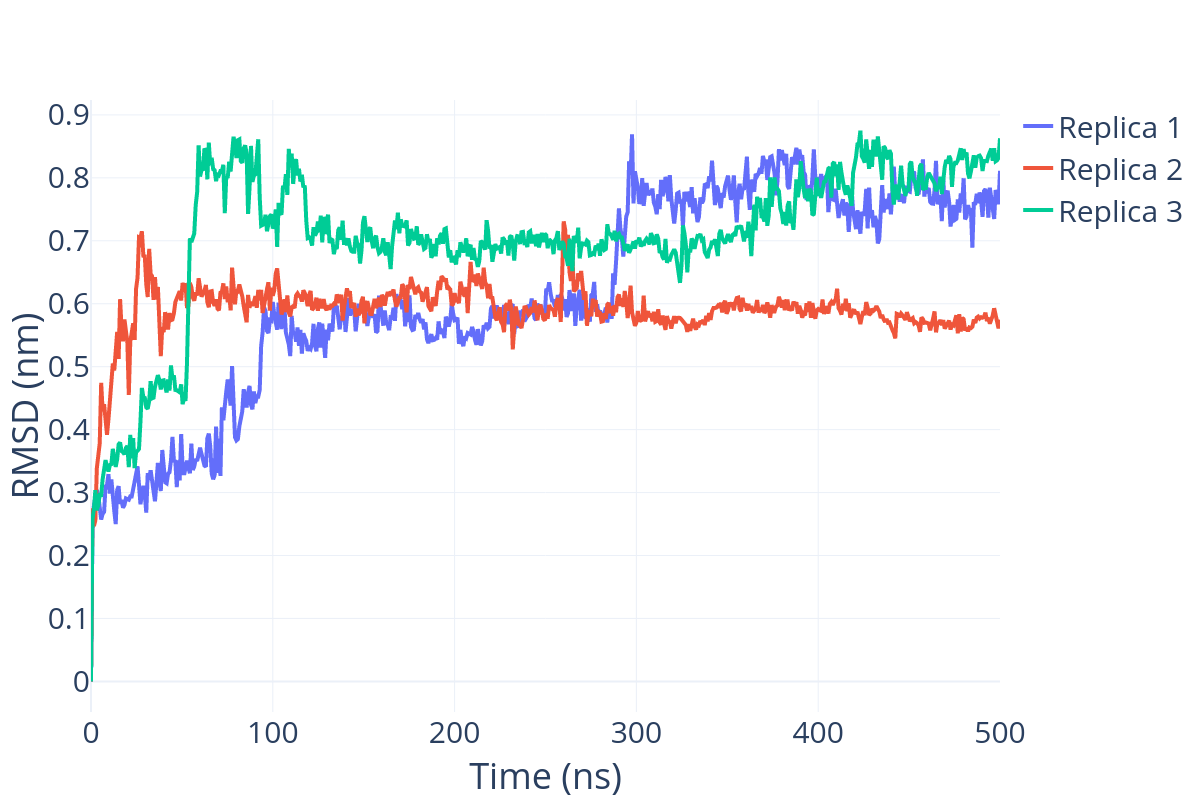


**Figure S8** Root mean square deviation (RMSD) of the PAS2 domain within the PAS1-PAS2 segment of human PASK, showing calculations for each replica. The RMSD fit was done over the backbone atoms of the whole segment and calculated for the whole protein (heavy atoms within the backbone and sidechains.


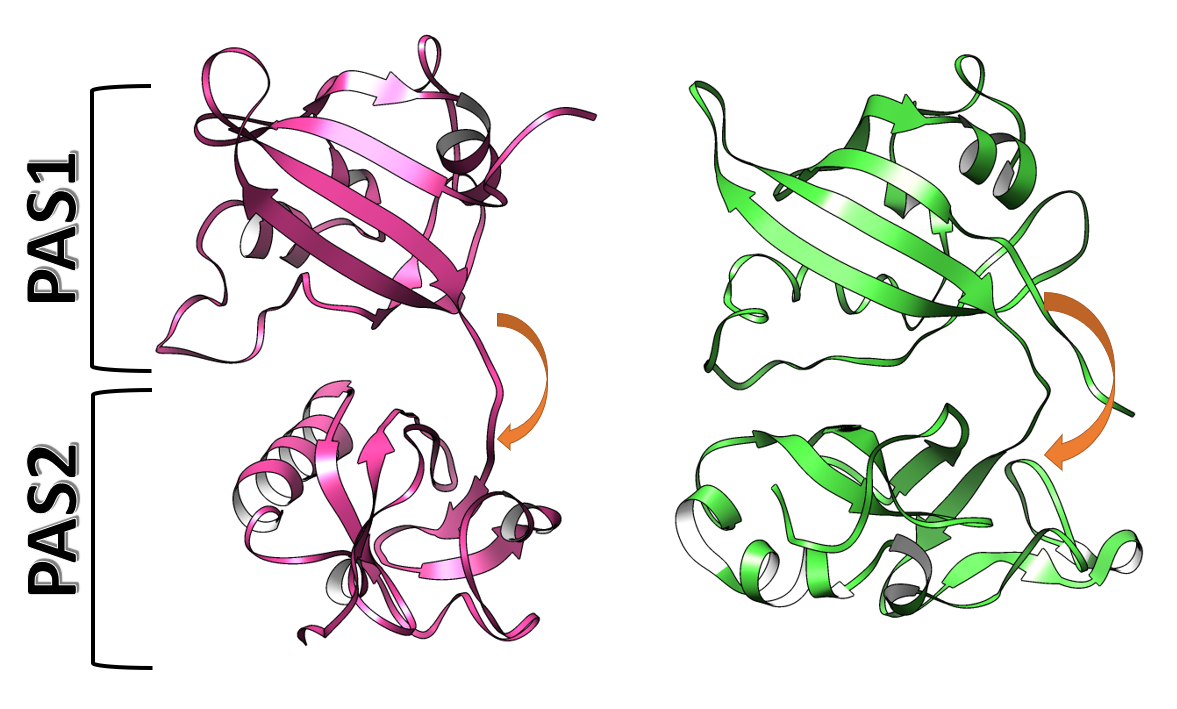


**Figure S9** Intrinsic dynamics of the short linker joining PAS1 and PAS2 domains. The starting conformation of the segment (residues 131-342) is coloured purple; the average configuration sampled throughout the 500 ns MD simulation is coloured green. The arrow indicates the linker region.


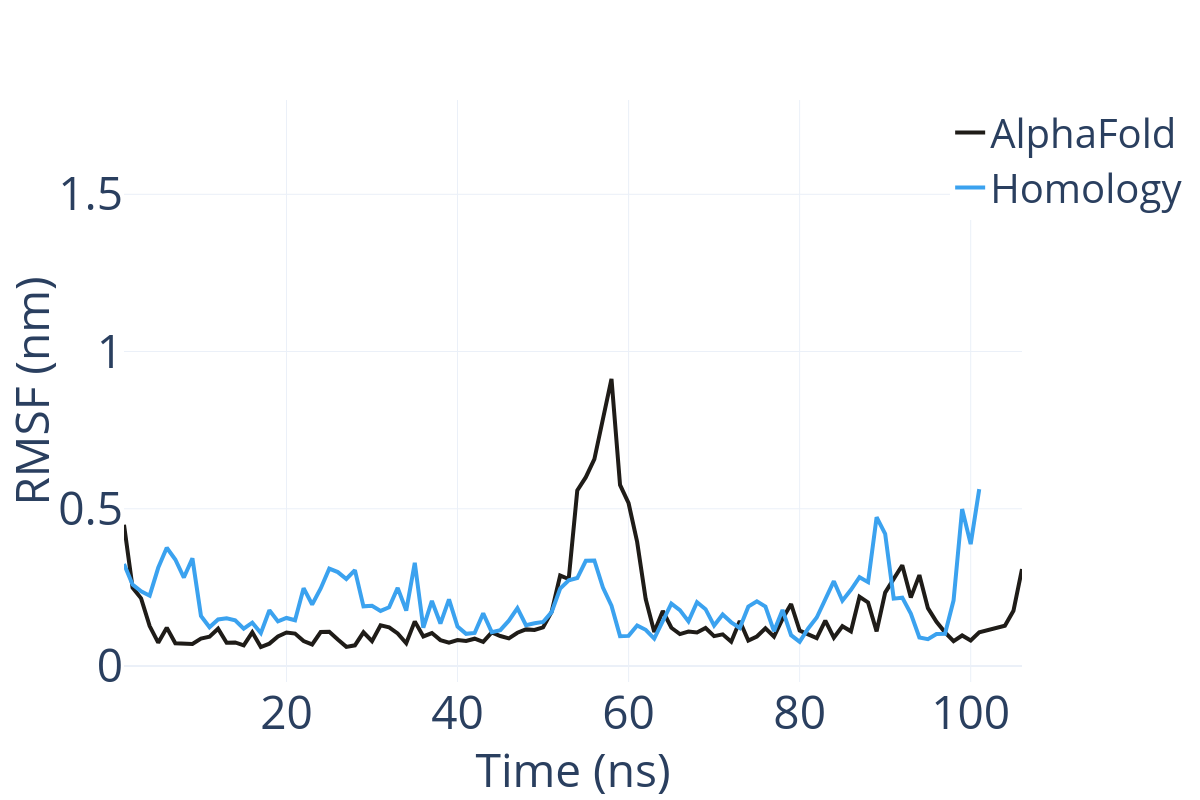


**Figure S10** Per-residue root mean square fluctuations (RMSF) calculated for models of PAS3 domain obtained by AlphaFold (black) and Phyre2 homology modelling blue). Residue index is adjusted to the structural alignment.

**
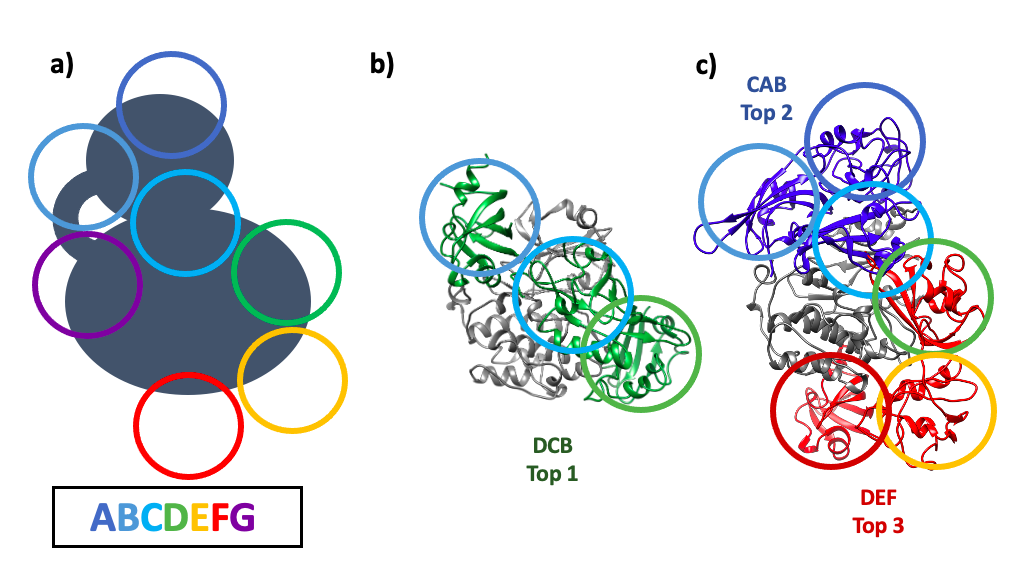
**

**Figure S11** **a)** Consensus positions of PAS domains of human PASK mapped in protein-protein docking. Protein kinase domain is represented as dim grey diagram, showing upper and lower lobes, connected by the hinge. Three PAS domains were mapped in regions represented as circles A-G. **b)** Best-scored model of PAS-kinase domain complex of PASK. The kinase domain is showed as backbone and coloured dark grey, the PAS1-PAS3 segment is coloured green.PAS1 domain is circled green and corresponds to the consensus position D; PAS2 domain is circled aqua and corresponds to the consensus position C; PAS3 domain is circled blue and corresponds to the consensus position B. **c)** Second-and third-best-scored models of PAS-kinase interactions. The kinase domain is showed as backbone and coloured dark grey, the PAS1-PAS3 segment are coloured blue (second-best model) and red (third-best model). Consensus positions of each of PAS domains are showed using the same rules as in the panel b.

| **Score** | **Sequence PAS1-PAS2-PAS3** |  | **Score** | **Sequence PAS1-PAS2-PAS3** |
| --- | --- | --- | --- | --- |
| 1 | **D-C-B** |  | 11 | A-B-G |
| 2 | **C-A-B** |  | 12 | B-C-D |
| 3 | **D-E-F** |  | 13 | B-C-A |
| 4 | G-C-B |  | 14 | B-A-C |
| 5 | G-C-D |  | 15 | A-C-D |
| 6 | D-C-G |  | 16 | C-D-E |
| 7 | D-C-A |  | 17 | C-B-A |
| 8 | G-B-A |  | 18 | F-E-D |
| 9 | B-C-G |  | 19 | E-D-C |
| 10 | A-B-C |  | - | - |

**Table ST1** Ranking of best-scoring 19 models of PAS-kinase domain complexes, predicted by ClusPro. Consensus positions indicate orientation of each PAS domain (PAS1, PAS2, and PAS3) relatively to the kinase domain. These consensus positions are aligned with the positions showed in Fig. S11 a).

| **Domain** | | | **Remarks** |
| --- | --- | --- | --- |
| **PAS1** | **PAS2** | **PAS3** |  |
| D190 | E236* | T350 | *PAS1-PAS2 linker |
| H192 | R237* | L352 | *PAS1-PAS2 linker |
| T199* | Q282 | H358 | *Via PAS1-PAS2 interactions |
| V200 | H289 | C343* | *PAS2-PAS3 linker |
| D202* | K292 | S346 | *Via PAS1-PAS2 interactions |
| S214 | R298 | N361 |  |
| W216 | R304* | F364 | *via PAS2-PAS3 |
| K218 | D305 | E916 | *via PAS2-PAS3 |
| R219 | K314* | Q918 | *via PAS1-PAS2 |
| Q222 | K316* | R913 | *via PAS1-PAS2 |
| E223 | T325 | E911 |  |
| E233 | T326 | W927 |  |
| - | E328 | - |  |

**Table ST2** Residues in putative PAS domains of human PASK that are likely to be involved in modulation of the kinase domain.

| **Domain** | **Total**  **Sites** | **PPI sites**  **(Y/N)** | **Consensus**  **hotspots** | **Consensus probe type** | **Representative probes** |
| --- | --- | --- | --- | --- | --- |
| PAS1 | 15 | Y | H1  H2  H3 PPI | Aromatic “drug-like”  Aliphatic hydrophobic  Hydrophilic  Mixed “drug-like” | 2-bromopyridine,  2-aminopyridine  n-hexane  Fructose, glucose, metformin,  pyrrolidin-2-ylmethanol,  cyclobutylboronic acid |
| PAS2 | 7 | Y | H4 PPI  H5 | Hydrophilic charged  Alicyclic hydrophobic | Metformin  Arginine  Glycerol-3-phosphate  Cyclohexanone |
| PAS3_AF | 11 | Y | H6  H7  H8 PPI  H9 PPI | Aliphatic hydrophobic  Aromatic “drug-like”  Alicyclic mixed  Positively charged  Hydrophilic | n-hexane  Phenol, 2-aminopyridine, 4-methylimidazole, 2-bromopyridine  Cyclohexanone, cyclobutanecarboxamide  Lysine, arginine, histidine, 4-methylimidazole  Glycerol, 2-cyclopropylethanol, Glycerol-3-phosphate |
| PAS3_h | 10 | Y | H10 PPI  H11  H12 | Hydrophilic  Hydrophilic  Positively charged | Glucose, lysine, pyrrolidin-2-ylmethanol  Glycerol, pyrrolidin-2-ylmethanol, arginine, piperazine, glycerol-3-phosphate, fructose  Histidine, 4-methylimidazole, lysine |
| Kinase/  PAS1-PAS3 | 4 | Y | H13 PPI  H14 PPI  H15 PPI | Aromatic “drug-like”  Positively charged  Hydrophilic | 2-aminopyridine, 2-bromopyridine  Metformin, arginine  Glucose, fructose, glycerol-3-phosphate |

**Table ST3** Consensus hot-spots identified in human PASK. PAS3_AF denotes AlphaFold model of PAS3 domain, while PAS3_h denotes PAS3 model calculated by the homology modelling (Phyre2).


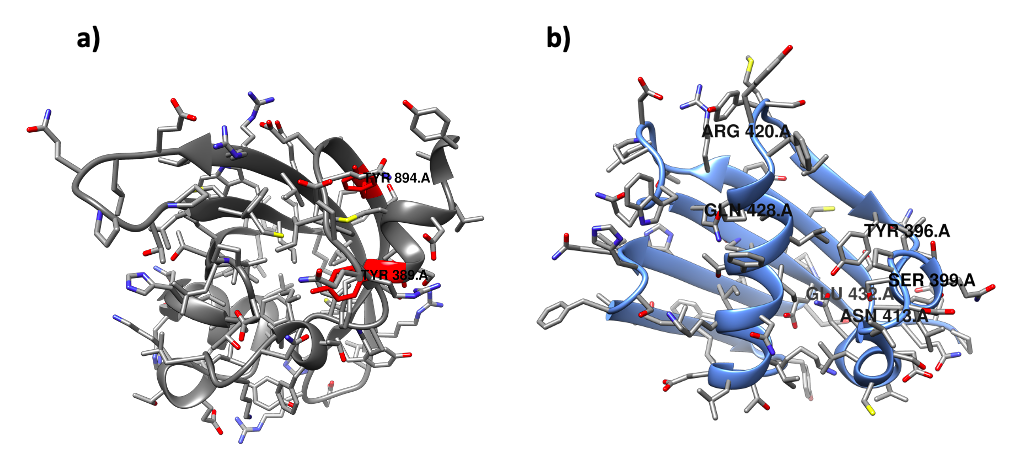


**Figure S12** A comparison between inner cavities on PAS3 models calculated by AlphaFold (**a)** and homology modelling (b). All residues are showed and coloured by element: oxygen – red; nitrogen – blue, sulphur – yellow. Residues capable of H-bond formation are labelled.


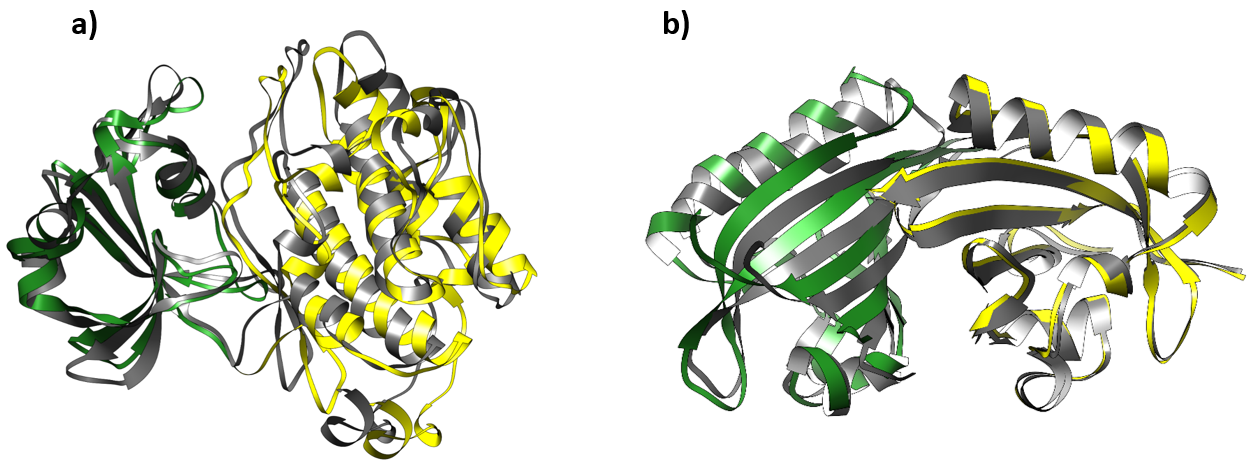


**Figure S13** Superimposed complexes resulting from protein-protein docking (grey) and respective crystal structures (green and yellow): (a) PDB code: 3DLS) and (b) PDB code: 3F1N. Both crystal structures were split into two segments which were coloured green and yellow, respectively. RMSD value for (a) is 1.216 (2.651 for all pairs) Å, for (b) is 0.904 (2.369 for all pairs) Å.


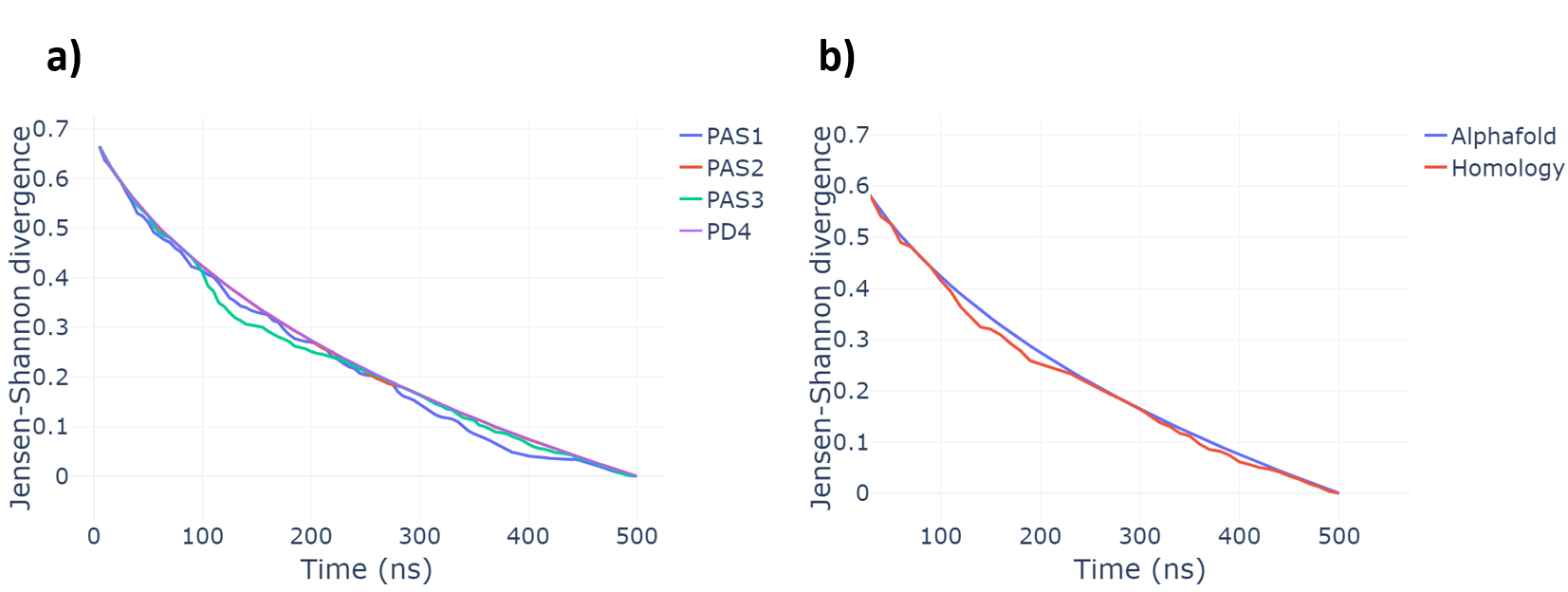


**Figure S14** Convergence plots of MD simulations a) For PAS1 (blue), PAS2 (red), PAS3 (cyan), and PAS4 (purple). b) For Alphafold PAS3 model (blue), and homology PAS3 model (red). Plots were made by MDAnalysis toolkit.


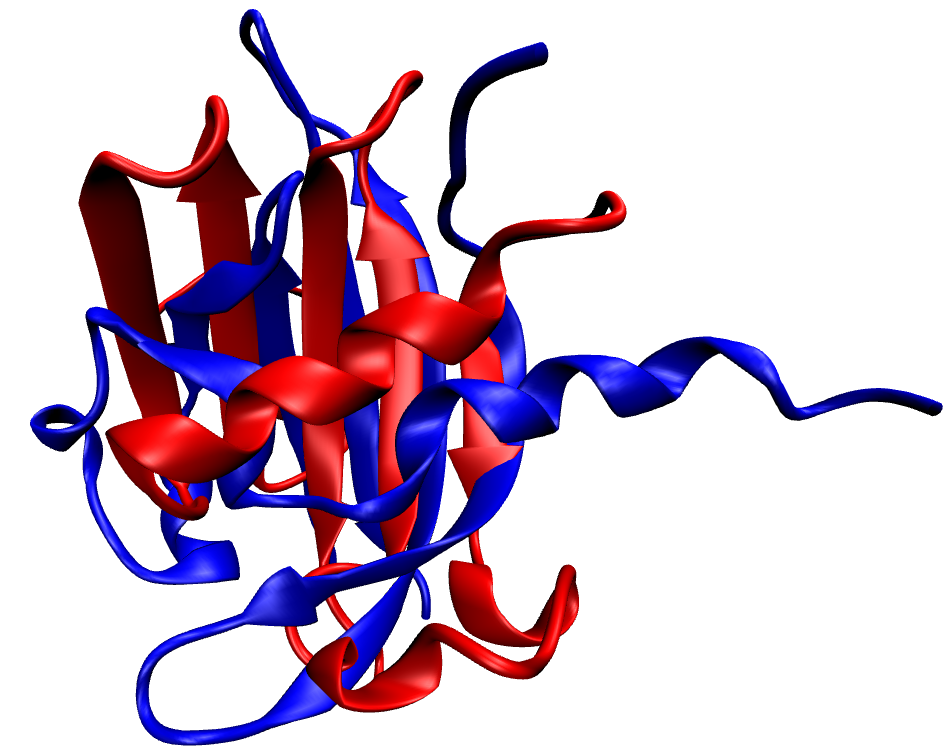


**Figure S15** Superimposed models of PAS3 domain calculated by Alphafold (blue) and homology modelling (red). RMSD = 14.4 Å. The figure was made by VMD RMSD analysis tool.

***Figure S16*** *Linear regression plot showing the correlation between the experimental dissociation constants Kd (x-axis) and calculated Kd values (y-axis) obtained for 6 small molecular inhibitors (Amezcua et al., 2002) binding to PAS1 domain of PASK. P value = 0.0029, R^2^ = 0.9136.*

| **Comp** | ***K_d_ ^exp^* (uM)** | ***K_d_ ^calc^* (uM)** |
| --- | --- | --- |
| **1** | 13 | 39.6 |
| **2** | 24 | 34.1 |
| **3** | 43 | 170.1 |
| **4** | 97 | 321.6 |
| **5** | 357 | 2465 |
| **6** | 1163 | 4111 |

***Table ST4*** *The experimental Kd values for the 6 compounds (Amezcua et al. (2002) binding to PAS1 domain of human PAK and their respective docking scores.*
